# PIPEQ-OS – an instrument for on-site measurements of the experiences of inpatients at psychiatric institutions

**DOI:** 10.1186/s12888-015-0621-8

**Published:** 2015-10-06

**Authors:** Oyvind Bjertnaes, Hilde Hestad Iversen, Johanne Kjollesdal

**Affiliations:** Department for Quality and Patient Safety, The Norwegian Knowledge Centre for the Health Services, Boks 7004 St Olavs Plass 0130, Oslo, Norway

## Abstract

**Background:**

The Psychiatric Inpatient Patient Experience Questionnaire (PIPEQ) was developed for post-discharge measurements of experiences, but the low response rates associated with post-discharge surveys restrict their usefulness. A new questionnaire was developed based on the PIPEQ for on-site measurements of patient experiences: the PIPEQ-OS. The aim of this study was to psychometrically test the PIPEQ-OS using data from a nationally representative survey conducted in Norway in 2014.

**Methods:**

Data were collected using a nationally representative patient-experience survey; 25 % of the institutions in each of the 4 health regions in Norway were randomly selected, yielding a total of 26 institutions. The PIPEQ-OS questionnaire was completed by patients on-site on an agreed day in week 37 of 2014. Item missing and ceiling effects were assessed, and factor analysis was used to assess the structure of the items included in the PIPEQ-OS. The scales were tested for internal consistency reliability, test–retest reliability and construct validity.

**Results:**

The initial sample comprised 857 patients. Of these, 60 were excluded for ethical reasons and 57 were excluded because they were absent on the day of the survey. Of the remaining 740 patients, 552 (74.6 % of the included population) returned the questionnaire. Low levels of missing or “not applicable” responses were found for 18 of the 21 items (<20 %), and 20 of 21 items were below the ceiling-effect criterion. Psychometric testing identified three scales: structure and facilities (six items), patient-centred interaction (six items) and outcomes (five items). All scales met the criterion of 0.7 for Cronbach’s alpha (range: 0.79–0.91) and test–retest reliability (range: 0.83–0.84). The construct validity of the scales was supported by 14 of 15 significant associations with variables known to be related to psychiatric inpatient experiences.

**Conclusions:**

The PIPEQ-OS comprises three scales with satisfactory internal consistency reliability and construct validity. This instrument can be used for on-site assessments of psychiatric inpatient patient experiences, but further research is needed to evaluate its usefulness as basis for external quality indicators.

## Background

Patient centredness is an important aspect of the quality of health care; it is a separate component of many health-care quality conceptualizations [1, 2] and is often measured by means of surveys of patient-reported experiences. Legislation and policy documents in many Western countries underpin this development, with an increased focus on patient rights and patient centredness. The proven link between patient experiences and clinical safety and effectiveness [3] has provided a clinical rationale for the focus on improving patient experiences. Numerous research studies and national quality measurement efforts have been conducted relating to patient satisfaction and patient experiences [4, 5]; however, the psychiatric services have not generally been included in this general literature. For example, psychiatry was excluded from a large systematic review of the measurement of patient satisfaction [4], and was only a minor part of the identified studies in a systematic review of the links between patient experiences and clinical safety and effectiveness [3]. Furthermore, a systematic review of national and cross-national surveys on patient experiences yielded only a few relevant surveys [5]. Thus, the general literature appears to offer relatively little insight into the measurement of the experiences of patients using psychiatric services.

Several specific reviews have been published on patient satisfaction with psychiatric services [6–9]. A review by Boyer et al. identified 15 self-report instruments that can be used to measure psychiatric inpatient satisfaction, based on a search of MEDLINE for the years 1988–2008 [9]. The review gave a rather negative view of existing instruments, stressing the lack of standardized definitions and measurement methods, and the inconsistent use of validation procedures [9]. Several instruments have since been published [10–13], but although it is possible that other instruments exist, the review and recently published primary studies point to a need for a standardized definition and measurement methods. This is supported by the conclusion of a general review of patient satisfaction instruments [14].

The Psychiatric Inpatient Patient Experience Questionnaire (PIPEQ) was developed and validated to measure patient experiences post-discharge [15, 16]. This questionnaire is part of the Norwegian program for the measurement of patient-reported experiences that was set up to provide external indicators at the institution level to support quality improvement, hospital management, free patient choice and public accountability. The PIPEQ was developed as part of the national program, but the very low rate of response to mailed post-discharge surveys of psychiatric inpatients restricts their validity and usefulness. A national survey performed in 2005 had a response rate of 35 % [17], and another survey conducted at a university hospital had a response rate of 23 %, even after two postal reminders [18]. In general, although the response rate is a poor indicator of non-response bias [19], the small number of patients per psychiatric institution in Norway renders it almost impossible to compensate for it by increasing the sample size.

The literature documents effective initiatives for increasing the response rate in postal and electronic surveys [20], but some of these have not proven successful when actually applied (e.g. multiple reminders) [18]. Furthermore, reviews of patient-satisfaction studies with psychiatric services have revealed that response rates can be higher in on-site studies than in post-discharge surveys [7, 9]. This finding was supported by a review of response rates in 210 patient-satisfaction studies, which found higher response rates for face-to-face recruitment than for mail surveys [21]. This has prompted a fundamental change to data collection, from post-discharge to on-site. Naturally, the content of the questionnaire had to be adjusted to the on-site context, and standardized procedures for data collection had to be developed.

The primary aim of this study was to psychometrically test the on-site version of the PIPEQ version (PIPEQ-OS) using data from a nationally representative survey conducted in Norway in 2014. While the study was not designed to assess the effect of on-site versus post-discharge data collection on the response rate, the response rate was clearly an important success criterion for the project.

## Methods

The national survey was conducted by the Norwegian Knowledge Centre for the Health Services (NOKC), commissioned by the Norwegian Directorate of Health, and was formally connected to the national quality indicator system, which meant that the selected institutions were obliged to participate.

### Data collection

The population consisted of adult (age ≥18 years) inpatients receiving specialized mental health care in 2014. Outpatient clinics, day units, age-psychiatric institutions, interdisciplinary treatment institutions for substance dependence and safety departments were excluded. Twenty-five percent of the institutions in each of the 4 health regions in Norway were randomly selected, yielding a total of 26 institutions. The sample comprised all patients staying at one of these institutions on an agreed day in week 37 of 2014.

The NOKC established regional contact persons to help compile the institution lists and to make contact with selected institutions. Two contact persons were established for each participating institution: a project-responsible professional and a substitute. The contact person at the institution informed staff about the survey, ensured that the institution complied with the recommended survey guidelines, sent out information regarding administrative data, including department overview, and ensured that a day for survey completion was selected. The institution contacts were responsible for establishing a member of staff for each department (the departmental responsible professional) who would be responsible for conducting the survey in that department. Tasks included disseminating information to the patients and employees, distributing and collecting of questionnaires, and reporting to the NOKC regarding the progress of the survey.

Standardized guidelines for data collection were developed. Each patient’s clinician was not allowed to be involved in the data collection. The patients were requested to complete the questionnaire by themselves, without discussion or influence from other patients or employees. The department employees were allowed to read to and help the patients to understand the questions, but without influencing the response. The departmental responsible professional distributed a closed envelope containing the questionnaire, an information letter regarding the survey and a reply envelope to the patient, and then collected the reply envelope containing the questionnaire when the patient had responded.

### Questionnaire

The PIPEQ-OS was based on the PIPEQ [15], but the question formulations were altered to account for the patients answering them on-site rather than post-discharge. Furthermore, since the PIPEQ was developed more than 10 years ago, it was necessary to update it according to the latest developments of the national program, which included the ten-item Generic Short Patient Experiences Questionnaire [18], a three-item modified version of the Patient Enablement Instrument (PEI) [22], and a newly developed patient-experience questionnaire for interdisciplinary treatment institutions for substance dependence [23]. The questionnaire was also updated according to the latest developments regarding layout, formatting and structure. The resulting PIPEQ-OS included most of the patient-experience questions from the PIPEQ, with adjustments made to four questions to ensure that it was concordant with the national program. A single item regarding activities was reformulated and supplemented with items regarding activities and facilities from the substance-dependence questionnaire [23], which have been identified in patient-based instruments as being important for psychiatric inpatients [11, 13]. A single item regarding improvement of mental health was replaced with the three-item modified version of the PEI, which has previously been included and tested in the patient-experience questionnaire for substance dependence [23]. This entire process resulted in the development of the first version of the PIPEQ-OS.

The PIPEQ-OS questionnaire was tested using cognitive interviews among psychiatric inpatients. Ten inpatients from three community mental health centres in one of the health regions in Norway were interviewed. The patients confirmed both the usefulness of responding on-site and the relevance and usefulness of questions on activities and facilities. Some patients reported general problems with reading and responding to the questionnaire, supporting the use of employees to read to and help patients to understand questions where necessary. Some adjustments were made after the interviews, but in general the cognitive interviews showed that the questionnaire functioned well and that the questions and topics were relevant to the patient group.

The revised version of the PIPEQ-OS comprised 41 closed-ended items. Most experience items had a 5-point response format ranging from 1 (“not at all”) to 5 (“to a very large extent”); 21 items related to structures, processes and outcomes at the institution were included in the psychometric testing.

### Statistical analysis

Items were assessed for missing data and ceiling effects, and factor analysis was used to assess the underlying structure of the items. Items with >20 % missing data were excluded from the factor analysis to avoid extensive loss of responses. Exploratory factor analysis was used to assess the underlying structure of the included items [24]. Principal-axis factoring and extracted factors with eigenvalues above 1 were used. Oblique rotation with Promax was used as a rotation method. Items with low factor loading were considered for removal, and items loading on several factors were placed in the most relevant theoretical factor. Two factor analysis was conducted, one among items of a structural or process character, and one on outcome items. The latter was conducted separately to avoid contamination from the process and structure variables and to test the unidimensionality of the outcome scale. The original authors found that the PEI and satisfaction were related, but were separate constructs [22].

The ceiling effect is defined as the percentage of respondents ticking the most favourable response option, and is an indication of potential problems with measuring changes over time and differences between providers. The criterion relating to the ceiling effect was set to 50 % [25, 26].

Items with poor factor loadings were considered for removal from the final solution. The internal consistency reliability of the resulting scales was assessed based on the item-total correlation and Cronbach’s alpha values. The former measures the strength of the association between an item and the remainder of its scale, while the latter assesses the overall correlation between items within a scale; a scale is generally considered to be sufficiently reliable when the alpha value is at least 0.7 [27]. The test–retest reliability was assessed by supplying every fourth patient with a retest questionnaire to answer approximately 2 days after completing it the first time. The level of agreement between the two sets of scores was assessed using the intraclass correlation coefficient.

Construct validity is concerned with the extent to which an instrument relates to other variables consistent with theoretically derived hypotheses concerning the constructs that are being measured. Construct validity was assessed through correlations of scale scores and comparisons with responses to five additional questions included within the questionnaire. Correlations between continuous background questions and the scales were assessed by Pearson’s *r*, and with *t*-tests for categorical questions. It was hypothesised that the scale scores would be correlated with civil status [11, 17] and previous admissions [11, 13], the reported experiences and satisfaction are generally worse for single patients and psychiatric patients with previous admissions. Furthermore, it was hypothesised that the scores would be associated with the level of coercion related to admission and/or treatment, because patients perceiving coercion report worse experiences and satisfaction [11, 13, 15, 28, 29].

All analyses were performed using SPSS (version 22.0).

### Approval

The national survey was conducted as an anonymous quality assurance project. According to the joint body of the Norwegian Regional Committees for Medical and Health Research Ethics, research approval is not required for quality assurance projects. The Norwegian Social Science Data Services states that anonymous projects are not subject to notification. Patients were informed that participation was voluntary and they were assured of anonymity. Vulnerable patients were protected by allowing the responsible professional at the institution to exclude individual patients for special ethical reasons. Return of the questionnaire represented patient consent, which is the standard procedure in all national patient experience surveys conducted by the NOKC.

## Results

Of the 857 patients who were initially included in the sample, 60 were excluded for ethical reasons and 57 were excluded because they were absent on the day of the survey. The questionnaire was completed and returned by 552 of the remaining 740 patients (74.6 % of the included population). As indicated in Table 1, 57.6 % of the respondents were female, 41.8 % were in the age range 25–44 years, 71.7 % were single, 28.2 % had never been admitted before, 26.6 % had a university or college education, and 11.4 % and 28.6 % reported very poor or poor mental health, respectively.

**Table 1:** Respondent characteristics (*n* = 552)

	*n*	%
Gender		
Female	292	57.6
Male	215	42.4
Age (years)		
18–24	90	17.8
25–44	212	41.8
45–66	180	35.5
≥67	25	4.9
Marital status		
Married	91	18.0
Cohabitating	52	10.3
Single	363	71.7
Education		
Primary school	116	23.0
Secondary school	254	50.4
University or college	134	26.6
Previous admissions		
0	143	28.2
1	91	17.9
2	49	9.7
3–5	93	18.3
>5	131	25.8
Self-perceived mental health		
Very poor	58	11.4
Rather poor	145	28.6
Both-and	160	31.6
Rather Good	104	20.5
Very good	40	7.9
General condition today		
Very poor	48	9.5
Rather poor	84	16.6
Both-and	190	37.6
Rather good	138	27.3
Very good	45	8.9
Self-perceived physical health		
Excellent	26	5.2
Very good	69	13.7
Good	182	36.2
Rather good	136	27.0
Poor	90	17.9

Table 2 indicates that 18 of the 21 items in the PIPEQ-OS instrument had low levels of missing or not applicable responses (<20 %); exceptions were the items regarding next of kin (36.9 %), medications (27.0 %) and discharge (25.4 %). Furthermore, 20 of the 21 items were below the 50 % criterion for the ceiling effect, with the exception being the question regarding malpractice (53.6 %).

**Table 2:** Item descriptions and characteristics

	*n*	Missing (%)	Not applicable (%)	Mean^a^	Ceiling (%)
Were you welcomed satisfactorily when admitted to the institution?	525	2.5	2.4	4.04	38.1
Have you had enough time for talks and contact with clinicians/personnel?	478	12.1	1.3	3.63	16.5
Do you perceive that the clinicians/personnel understand your situation?	481	12.0	0.9	3.64	22.9
Have you had the chance to tell the clinicians/personnel what is important about your condition?	475	12.1	1.8	3.70	20.2
Do you consider that the clinicians/personnel have cooperated well with your next-of-kin?	348	12.1	24.8	3.19	15.5
Do you consider that the clinicians/personnel have prepared you for the time after discharge?	412	12.9	12.5	2.75	7.3
Do you consider that your treatment has been adjusted to your situation?	472	12.0	2.5	3.55	17.4
Have you had influence on the choice of treatment regime?	457	12.3	4.9	2.88	8.8
Have you had influence on your medication?	403	12.3	14.7	3.04	16.1
Has the institution given you adequate information about your mental condition/diagnosis?	447	12.3	6.7	3.13	12.1
Has the institution given you adequate information about the treatment options available to you?	444	12.7	6.9	2.85	9.0
Have you felt safe at the institution?	475	12.7	1.3	4.05	37.5
Have the activities offered at the institution been satisfactory?	457	13.2	4.0	3.59	22.8
Have the meals at the institution been satisfactory?	521	4.5	1.1	3.86	28.8
Have you been satisfied with the possibility for privacy?	515	5.4	1.3	3.77	25.6
Do you believe that you have been subjected to malpractice during your stay (based on your own opinion)?	513	5.1	2.0	4.12	53.6
Have the help and treatment you have received at the institution improved your ability to understand your mental condition?	502	4.9	4.2	3.41	20.5
Have the help and treatment you have received at the institution improved your ability to cope with your mental condition?	505	5.1	3.4	3.24	12.1
Have the help and treatment you have received at the institution led you to believe that your life will improve after discharge?	504	5.3	3.4	3.30	16.1
All in all, have the help and treatment you have received so far at the institution been satisfactory?	525	4.9	-	3.61	18.1
All in all, what benefit have you gained from the treatment you have received so far at the institution?	514	5.1	1.8	3.33	14.0

^a^All items were scored on a 5-point response scale ranging from 1 (“not at all”) to 5 (“to a very large degree”).

Seventeen items were included in the factor analysis. The first factor analysis included 12 items related to structure and process and resulted in 2 factors with an eigenvalue of >1 that explained 55 % of the variation: 1 related to structure and facilities and 1 related to patient-centred interactions (Table 3). The second factor analysis included five outcome items and resulted in one factor with eigenvalue of >1; this factor explained 74 % of the variation. All scales met the criterion of 0.7 for Cronbach’s alpha: the alpha values for structure and facilities, patient-centred interaction and outcome were 0.79, 0.86 and 0.91, respectively; the corresponding test–retest reliabilities for these three parameters were 0.84, 0.83 and 0.84.

**Table 3:** Factor loadings and reliability statistics

	Factor loadings^a^	Item-total correlation	Cronbach’s alpha	Test–retest reliability
*Structure and facilities:*			0.79	0.84
Were you welcomed satisfactorily when admitted to the institution?	0.63	0.60		
Have you had enough time for talks and contact with clinicians/personnel?	0.49	0.55		
Have you felt safe at the institution?	0.62	0.54		
Have the activities offered at the institution been satisfactory?	0.46	0.55		
Have the meals at the institution been satisfactory?	0.72	0.46		
Have you been satisfied with the possibility for privacy?	0.58	0.55		
*Patient-centred interaction:*			0.86	0.83
Do you perceive that the clinicians/personnel understand your situation?	0.52	0.73		
Have you had the chance to tell the clinicians/personnel what is important about your condition?	0.65	0.60		
Do you consider that your treatment has been adjusted to your situation?	0.52	0.74		
Have you had influence on the choice of treatment regime?	0.65	0.57		
Has the institution given you adequate information about your mental condition/diagnosis?	0.80	0.66		
Has the institution given you adequate information about the treatment options available to you?	0.79	0.61		
*Outcomes:*			0.91	0.84
Have the help and treatment you have received at the institution improved your ability to understand your mental condition?	0.79	0.75		
Have the help and treatment you have received at the institution improved your ability to cope with your mental condition?	0.88	0.83		
Have the help and treatment you have received at the institution led you to believe that your life will improve after discharge?	0.77	0.73		
All in all, have the help and treatment you have received so far at the institution been satisfactory?	0.84	0.79		
All in all, what benefit have you gained from the treatment you have received so far at the institution?	0.83	0.78		

^a^Separate factor analysis for outcomes.

The construct validity of the scales was supported by 14 of 15 significant associations with variables known to be related to the experiences of psychiatric inpatients (Table 4). The difference between single patients and those who were married or cohabitating was around 5 points on a scale from 0 to 100 (where 100 is the best score), with singles reporting worse experiences. Previously admitted patients reported worse experiences than patients without a previous admission, but one of three differences was not significant. There were large differences between patients who were admitted voluntarily and involuntarily (>10 points for all scales), with the former group reporting better experiences. Correlations between the scales and two other variables about perceived coercion were also significant and in the same direction as for voluntary versus involuntary admission.

**Table 4:** Construct validity testing: associations between scales and other variables

	Structure and facilities	Patient-centred interaction	Outcomes
Married or cohabitating	**	*	*
No	64.7	56.5	57.6
Yes	70.0	61.4	63.6
Previous admissions	*	ns	*
No	69.0	59.9	63.3
Yes	65.0	57.2	57.7
Voluntary admission	***	***	***
No	55.0	44.1	43.6
Yes	68.7	60.9	62.7
Admission perceived as necessary or unnecessary	0.28**	0.19**	0.20**
Perception of treatment coercion	0.44**	0.42**	0.49**

***p < 0.001**p < 0.01; *p < 0.05; ns, not significant. Pearson’s *r* for continuous variables.

## Discussion

The PIPEQ-OS comprises three scales with satisfactory internal consistency reliability, test–retest reliability and construct validity. Two of these scales concerns patient-reported experiences with structures and processes of inpatient care, while the third concerns patient evaluation of outcomes. The instrument was tested in a nationally representative survey in Norway, with a response rate of 74.6 % and low levels of item non-response, thus demonstrating the feasibility and acceptability of on-site data collection.

The PIPEQ-OS is multidimensional and comprised three scales, while the original PIPEQ for post-discharge measurement comprises only one scale [15, 17]. One reason for this difference was the decision to conduct two separate factor analyses. However, the additional scales were mainly the result of content changes in the questionnaire: outcomes was supplemented with three modified items from the PEI [22], while the on-site context warranted more questions on structure and facilities, since these aspects are important for inpatients [11–13]. The PEI measures a concept different from, but related to, patient satisfaction [22]. However, the PEI developers correlated the instrument with broad satisfaction instruments that included aspects such as the length of consultation and depth of relationship, which in our terminology relates to structure and process, and not outcomes. It is difficult to compare the dimensionality of the PIPEQ-OS with other inpatient psychiatry instruments since there is considerable variation in the number of dimensions [9–13]. However, the “structure and facilities” scale resembles the term “functional aspects” described in a large systematic review of patient experiences, while the “patient-centred interaction” scale resembles what was referred to elsewhere as “relational aspects” [3]. The PIPEQ-OS does not include questions about adverse effects or patient safety, but might easily be supplemented with validated patient safety instruments if they form part of the study topic (e.g. the Experiences of Therapy Questionnaire) [30].

The PIPEQ-OS is one of many instruments that have been developed for measuring patient evaluation of psychiatric inpatient care. A review of satisfaction instruments published in 2009 identified 15 instruments, but found an absence of a unified measurement approach [9]. Recently published instruments also exhibit a lack of a universal definition and measurement approach [10–13]. Conceptually, three studies claim to have assessed patient satisfaction [10–12], and one measured patient perceptions [13]; however, all of these studies varied regarding item generation methods, subthemes, number of questions and response scales. In fact, only one of these four studies refers to the review from 2009 [11], but the report of that study was co-authored by the first author of the review. Previous reviews also point to the lack of standardization and conceptual models [6–8]. The PIPEQ-OS was developed and validated as part of a long-standing national patient-experience program, consisting of around 15 standardized instruments for measuring patient experiences with different patient groups. This national program uses standardized development and validation methodology, data-collection procedures, type of questions, response scale, and scoring and case-mix system [31–36]. The questionnaire content is heavily based toward patient views. The conceptual approach for the program makes a distinction between patient-reported experiences with non-clinical issues, patient-reported safety and patient-reported outcomes, but also allows concurrent measurement of several components [37]. Furthermore, the conceptual approach draws on the work of Donabedian [38], and links patient-reported experiences to structures and processes of care, while patient satisfaction is considered an outcome. The PIPEQ-OS was developed to measure patient experiences on-site – not post-discharge – following data-collection procedures from a national patient-experience survey with substance-dependence institutions conducted in 2014 [23]. The three scales were interpreted according to the aforementioned terminology: the structure and facilities scale and the patient-centred interaction scale are conceptually linked to patient-reported experiences, while the outcomes scale is linked to patient-reported outcomes. Thus, the PIPEQ-OS has a clear and broad conceptual base, connecting evaluations from psychiatric patients to the tradition of patient-reported experiences [39], while simultaneously including an outcome scale that combines elements of the traditions of patient-satisfaction measurements [4] and patient-reported outcomes [40–42].

All of the PIPEQ-OS subscales have excellent psychometric properties and are relevant to use as a basis for identifying external quality indicators. However, it is important to note that single items of importance to patients should be retained in the questionnaire, including items regarding next of kin, medications and preparation for discharge, which were excluded following psychometric testing. These topics are important for many patients and are potentially useful for assessing quality improvement. For instance, in a study comparing experiences and importance across ten different patient groups, preparation for discharge was the worst-scoring experience item of all items for inpatient psychiatric patients [18]. Measurement properties are clearly worse for single items than for multi-item scales, but the single items are still an important part of the questionnaire.

More research is needed to evaluate the usefulness of using the PIPEQ-OS as an external quality indicator. This includes correlation studies between the PIPEQ-OS and other quality indicators, analyses that evaluate the discrimination between providers and sensitivity to detect changes over time, and development and testing of case-mix models to use in benchmarking. The predictive validity of the PIPEQ-OS should be studied, and particularly the ability of the outcomes scale to predict compliance and outcomes post-discharge. Qualitative studies to assess local implementation of the data collection protocol should be conducted since local variation might damage comparability. Research on the use of patient-experience data to improve services in this context is important [43], including barriers toward the use of such data, and factors that promote their use [44]. Lastly, the PIPEQ-OS might be cognitively demanding for some patients, and too long for studies with multiple measures. The possibility of obtaining a short version should be assessed in future research.

### Limitations

The PIPEQ-OS is part of a national program whose aim is to provide external indicators at the institution level, and to support quality improvement, hospital management, free patient choice and public accountability. While the PIPEQ-OS might function well as a basis for external quality indicators, some limitations should be mentioned. First, the number of responses per institution in this sample is too small to achieve robust quality indicators at the institution level. The choice of surveying all inpatients on a particular day was driven by economic and practical considerations, resulting in few responses and confidence intervals that are too wide to justify confirmation of external indicators at this level. For future national surveys it is recommended that all patients are surveyed within a 4- to 6-month period for each participating institution in order to obtain a sufficient number of responses for constructing external indicators, based on the current size of inpatient institutions in Norway. Second, the one-day approach means that patients are included at different phases of their treatment. While this can be handled with proper adjustment and cautious interpretation, interpretation of all scales might benefit from standardization of timing to the end of the inpatient stay. Data collection close to discharge is also the most common approach in the literature [9]. Third, on-site measurements have been shown to inflate patient evaluation ratings compared to mailed post-discharge surveys [16, 45–48], possibly causing problems with discriminating between providers and detecting changes over time. In a Norwegian study involving psychiatric inpatients, the overall on-site score was around 10 points more positive than the post-discharge score on a scale from 0–100, where 100 is the best [16]. The differences between the national surveys conducted in 2005 (post-discharge) and 2014 (on-site) were typically 5–10 points, depending on the item. Consequently, on-site measurements appear to result in the reporting of experiences that are too positive, implying that comparisons between surveys with different data collection modes should be avoided. However, the PIPEQ-OS produced ceiling effects that were much smaller than those observed in other studies [7]; thus, discriminating between providers and detecting changes over time should not be a major problem. Fourth, the outcome scale measures an intermediate outcome at a single point in time, and is based only on the patient perspective. This scale should be supplemented with other clinical quality indicators and perspectives, and more robust pre–post measurement of patient-reported outcomes. However, the current Norwegian quality indicator system lacks outcome indicators for mental health care. Thus, at the present time the POPEQ-OS has the potential to provide useful information regarding patient-reported outcomes in addition to patient-reported experiences. Lastly, no information was registered about non-respondents and reasons for exclusion based on ethical reasons, causing some uncertainty regarding the generalizability of results.

## Conclusions

The PIPEQ-OS comprises three scales with satisfactory internal consistency reliability and validity. The instrument can be used to assess the experiences of psychiatric inpatients on-site, but further research is needed to evaluate its usefulness as basis for external quality indicators.
